# Thermal Disorder in Finite-Length Carbon Nanowire

**DOI:** 10.3390/ijms24098149

**Published:** 2023-05-02

**Authors:** C. H. Wong, E. A. Buntov, W. S. Yip, S. To, M. B. Guseva, A. F. Zatsepin

**Affiliations:** 1 Department of Industrial and Systems Engineering, The Hong Kong Polytechnic University, Hong Kong; sandy.to@polyu.edu.hk; 2 Research Institute for Advanced Manufacturing, The Hong Kong Polytechnic University, Hong Kong; 3 Institute of Physics and Technology, Ural Federal University, 620002 Yekaterinburg, Russia; e.a.buntov@urfu.ru (E.A.B.); a.f.zatsepin@urfu.ru (A.F.Z.); 4 State Key Laboratory of Ultra-precision Machining Technology, Department of Industrial and System Engineering, The Hong Kong Polytechnic University, Hong Kong; 5 Faculty of Physics, Lomonosov Moscow State University, 125009 Moscow, Russia

**Keywords:** carbon nanowire, phase transition, Monte Carlo simulation

## Abstract

Enhancement in chemisorption is one of the active research areas in carbon materials. To remedy the thermally degraded chemisorption occurring at high temperatures, we report a comprehensive study of kink structures in free-standing monoatomic carbon nanowires upon heating. Our Monte Carlo simulation considers multi-monoatomic carbon chains laterally interacting by van der Waals forces. Our study reveals that carbon nanowires maintain their linearity regardless of chain length at low temperatures, but this is not the case at high temperatures. Disordered kink structure is observed in short carbon chains, especially above the Peierls transition temperature. A severe kink structure may increase the possibility of attaching negatively charged atoms, thereby contributing to the development of next-generation materials for chemisorption at high temperatures. We have also provided an important indication that any physical property of the finite-length carbon chain predicted by ab initio calculation should reconsider the atomic rearrangement due to thermal instability at high temperatures.

## 1. Introduction

Carbon exists in various configurations such as graphite, diamond, graphene, nanotubes, etc. The advantage of using carbon nanomaterials for chemisorption is their promising ability to provide a wide range of adsorption applications such as water treatments [1] and nanosensors [2]. For water treatments, the production of oil and gas generates a large amount of wastewater, known as produced water, which can contaminate soil and water resources if released into rivers and oceans. In response to this, treatment and management of the produced water is necessary to mitigate the environmental impact. Expanded graphite and activated carbon adsorbents have been studied as cost-efficient and effective means of treating this wastewater [1,3]. From sensing technology’s point of view, the response of in situ grown carbon nanotubes (CNTs) on a SiO_2_ substrate when supplied with a constant current has been observed as a voltage response with respect to time to detect gas concentrations of H_2_ and CO_2_. The adsorption and desorption curves of the voltage response were fitted to two different exponential behaviors, allowing characteristic times to be identified that separately indicated chemisorption and physisorption processes on the CNTs [2]. In addition, the low atomic number of carbon makes electron–electron interactions simpler than those of heavy metals, which can be beneficial for the development of kinetic and equilibrium models [3] to predict and design adsorption processes. In contrast, graphene and its composites have been demonstrated as promising adsorbents in high-temperature environments involving hydrogen, carbon dioxide, and methane in comparison to those traditionally activated carbons with large surface areas [4]. Hydrogenated graphene contains sp^3^ C-H bonds in the basal plane that can be used as hydrogen storage [4].

Porosity can be a key to enhance chemisorption. Charged particles can be bound more effectively with the help of the pores, which create a stronger local potential for the particles to attach to. This increased potential for particle attachment originates from a higher charge density, which leads to better absorption compared to non-porous materials. For example, porous graphene oxide-like foam provides the selective absorption of carbon dioxide over nitrogen, methane, hydrogen, and carbon monoxide at the conditions of 1 × 10^6^ Pascal and 300 K with the help of the aliphatic and aromatic domains with oxygen-rich functional groups on the surfaces [4]. Similarly, molecular absorption in porous carbon nanotubes is also expected after the nanotubes are electrostatically charged in ionic solutions [5]. The curvature-assisted effective atomic number Z (or local charge density) is proposed for thinner nanotubes, and may offer an alternative method to improve chemisorption [6]. A dielectrophoretic (DEP) fabrication method has been developed as a promising technique for the fabrication of carbon nanotubes (CNTs). This process, in combination with the right frequencies, enables the alignment of single-walled CNTs (SWCNTs), which is expected to have better sample quality [7,8]. By introducing porous sites on SWCNTs, it creates lattice distortions or local curvature to attract ‘flying’ atoms for chemisorption. If the carbon nanomaterials exist in the presence of numerous local curvatures, it may bring hope for effective chemisorption, even at high temperatures, due to the modified effective atomic number Z [5,6,9].

The pure 1D form of carbon, the monoatomic carbon chain, has become a hot research topic in recent years due to the massive elastic modulus, torsional-induced magnetism, and large electronic density of states at the Fermi level [10,11,12]. The monoatomic carbon chain may generate numerous local curvatures in the form of kinks owing to 1D atomic fluctuations, which may allow effective chemisorption. Linear-chain carbon contains two phases [13]. The metallic cumulene phase is likely more energetically favorable at low temperatures and the semiconducting polyyne phase should appear above the Peierls transition temperature (500 K) [13,14,15]. However, the synthesis of the linear chain of carbon presents a huge challenge. Using the most modern technology could only obtain about 6000 atoms of carbon nanowire, which unavoidably required protection from the double-walled carbon nanotube [11]. As chemisorption pales at high temperatures [2], we are trying to investigate whether the kinks will be generated in finite-length carbon nanowires upon heating. The disordered carbon chain associated with the kink structure may open opportunities for weakening the thermal effect of chemisorption.

## 2. Results and Discussion

**Figure 1:**
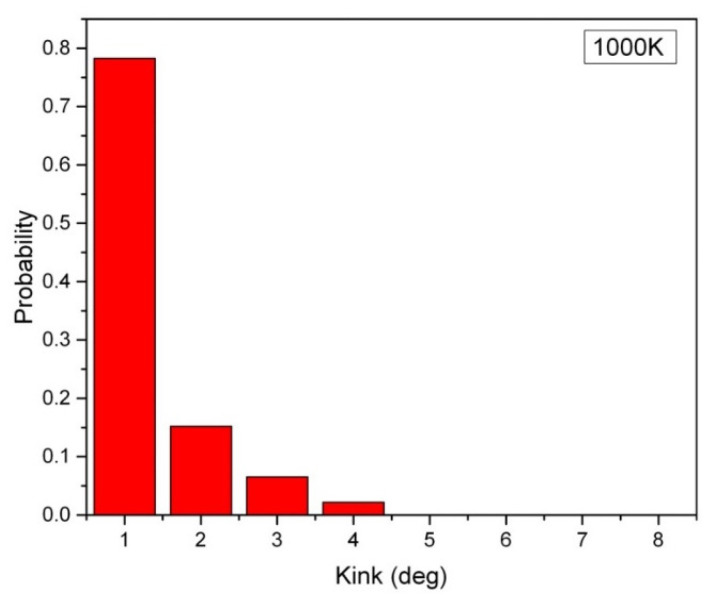
The distribution of kink angles in the 10 parallel 2000-CNA at 1000 K. Each nanowire carries 2000 atoms.

**Figure 2:**
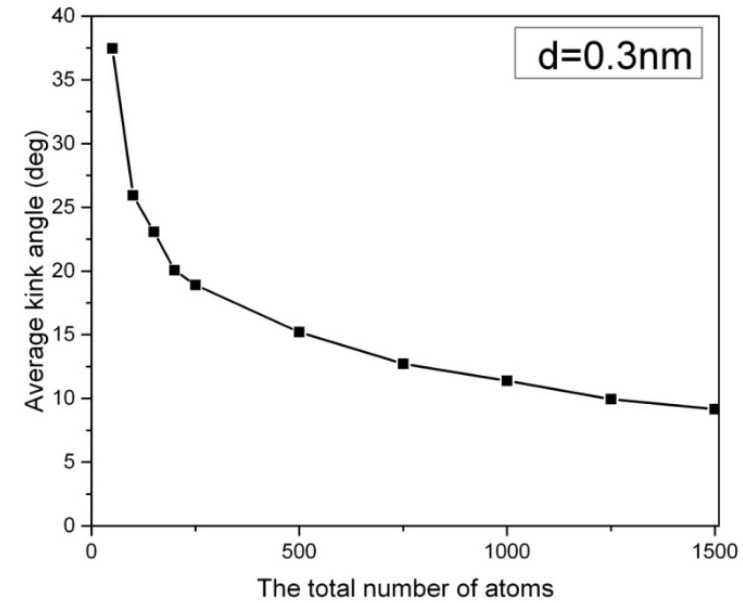
The average kink angle of the carbon nanowires array as a function of chain lengths at 1000 K. d is the lateral distance between the carbon chains.

In Figure 1, the distribution of the kink angle in a hexagonal array of carbon nanowires at 1000 K is presented, where the carbon nanowires are composed of 2000 atoms, and they have a lateral chain-to-chain distance of d = 0.3 nm. The most probable kink angle of 1 degree is observed, while larger kink angles also appear with exponentially decreasing probability. The average kink angle of the entire sample, which can be considered linear, is only 2.9 degrees. However, the kink structure becomes more pronounced in short carbon nanowires containing fewer than 250 atoms. Figure 2 depicts the average kink angle of carbon nanowires with different chain lengths, with short nanowires showing an average kink angle of 38 degrees in 50-CNA. This phenomenon can be attributed to the lack of atomic spring constants in short carbon nanowires, making it difficult to control the kinematics of any rapidly moving atom at high temperatures. In contrast, atoms displaced from their equilibrium position along infinitely long carbon chains always contend with numerous serial atomic spring constants; where the size of the chain increases, the atomic fluctuations decrease [16].

**Figure 3:**
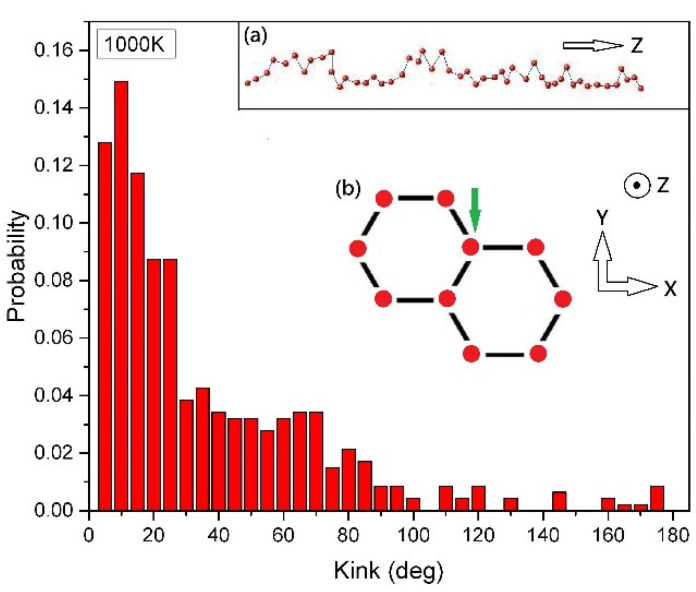
The distribution of the average kink angles in the 10 parallel 50-CNA at 1000 K. Each nanowire contains 50 atoms. Inset (**a**) depicts the side view of a carbon chain (marked by a green arrow) in the nanowire array at 1000 K, while inset (**b**) presents the side view of 10 parallel 50-CNA at the initial condition.

Figure 3 depicts the ten 50-CNA structures at a high temperature of 1000 K. Unlike the 2000-CNA, the 50-CNA exhibits a diverse range of kink angles that extend beyond 100 degrees, due to strong thermal fluctuations. The inset (a) of Figure 3 displays the atomic distribution of a carbon chain in 50-CNA at 1000 K. At such high temperatures, the atoms gain enough thermal energy to overcome the covalent interactions between carbon atoms and they start to vibrate around their equilibrium positions instead of forming a perfect linear alignment. This further disrupts the linear alignment of the carbon atoms and causes them to vibrate in lateral directions.

**Figure 4:**
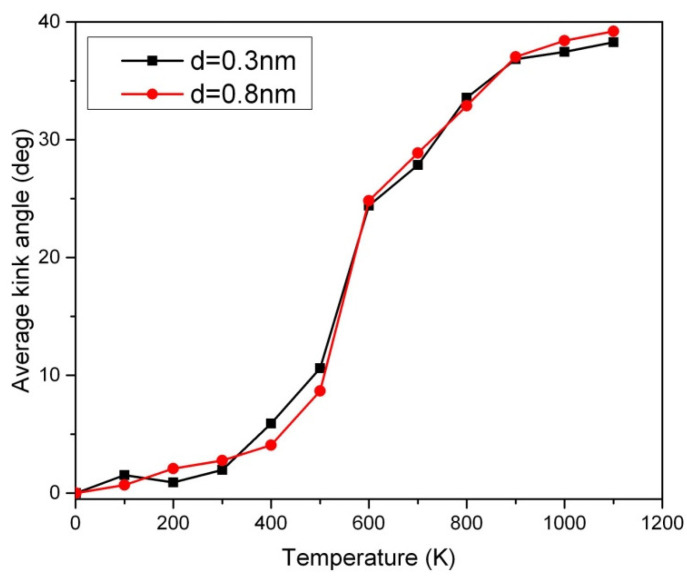
The thermal effect of the average kink angle of the 10 parallel 50-CNA in various van der Waals couplings, where d is the chain-to-chain distance.

The Peierls transition is a phenomenon that occurs in certain one-dimensional crystalline solids [13]. It involves a sudden change in the lattice structure at the Peierls transition temperature [13]. At its most basic level, the Peierls transition mainly results from a competition between two forces: the kinetic energy and lattice energy of the crystalline materials. When the lattice energy is strong enough, it will cause a structural rearrangement of the crystalline materials, resulting in a decrease in its overall conductivity (or opening a band gap as a semiconductor). In contrast, when the temperature is high enough, the fast kinematic motion of atoms will cause them to break away from the structural pattern they had formed at low temperatures, allowing them to return to the metallic state [11]. However, the carbon chain prefers to undergo an inverse Peierls transition in which it favors the metallic (semiconducting) state below (above) the Peierls transition temperature [13]. The Peierls transition of short carbon chains has been probed by our new method based on the temperature sweep analysis in Figure 4. The slope or inflection point in Figure 4 can be used for the detection of the Peierls transition. Below the Peierls transition temperature, no significant disorder in the 50-CNA was observed, as indicated by an average kink angle of less than 10 degrees. This is attributed to insufficient Boltzmann excitation in low-temperature regimes. However, a disordered kink structure is observed above the Peierls transition temperature [13,14] as evidenced by a sharper upturn at ~500 K in Figure 4. Interestingly, using the Boltzmann factor alone is not sufficient in explaining the rapid increase in the average kink angle across the phase transition. The Peierls instability appears to actuate the changes in the period of the one-dimensional nanowire that presumably forms kinks. Therefore, the rigorous atomic motion due to the Boltzmann excitation in combination with the increase in entropy across the Peierls transition causes carbon atoms to align more chaotically. The disorder in the 50-CNA starts to saturate above 800 K due to the stabilization of thermal oscillations [16].

The van der Waals force is a type of interaction between molecules or atoms that occurs due to the imbalance in the electron density across the molecule. This imbalance causes temporary dipoles in the molecule, which create an attraction between molecules [11]. However, these forces are weak and short-range, so they are not strong enough to make a significant impact on the atomic structure of the carbon chain, and so does the Peierls transition temperature, where the strong covalent bonds between the carbon atoms provide the dominant force in the chain [17,18]. In other words, van der Waals forces are not strong enough to influence the Peierls transition temperature as shown in Figure 4. Figure 4 also shows that the kink structure is non-negligible in short carbon nanowires above the Peierls transition temperature, which may enhance chemisorption at high temperatures.

The magnetic measurements of long carbon chains obtained with our SQUID magnetometer showed ferromagnetism up to at least 400 K [19]. To trace the origin of the unusually strong ferromagnetism, we applied an ab initio simulation, which showed that the ferromagnetism in these chains appeared only if the kink angle was less than ~10° [19]. Although the highest achievable temperature with the SQUID magnetometer was 400 K, the strong ferromagnetism of the carbon chains remaining intact at this temperature could be taken as an indirect indication that the kink angles of the chains below this temperature were small, which is consistent with the Monte Carlo results in Figure 4. Chemisorption can be affected by thermal energy. The frequency of chemisorption decreases as the temperature rises. This is due to the increased thermal motion of adsorbate particles, which reduces the effectiveness of interaction (covalent or ionic) during chemisorption. The increased surface vibration of the materials also makes the adsorbate particles more difficult to form bonds for chemisorption. In other words, the concentration of adsorbate particles is reduced upon heating. Can the kink-structuring carbon nanowire perform chemisorption effectively? Preliminary research on carbon nanotubes shows that the effective nuclear charge Z is increased due to curvature [6]. The kink structure in the short carbon chain creates numerous local curvatures and the effective Z is eventually expected to be enhanced. The average kink angle of about 40 degrees in the 10 parallel 50-CNA at 1000 K causes about a 30% increase in the linear charge density from the positively charged lattice [20]. It may increase the efficiency of absorbing negatively charged atoms at kinks, which opens an opportunity to absorb toxic gases from the environment [21,22]. DFT simulations are desired to predict the atomic fluctuations at 0 K. Unlike DFT simulations focusing on ground states, our Monte Carlo method is capable of creating thermal disorder of carbon nanowires in excited states at high temperatures, which allows the design of carbon nanowire-based devices for high-temperature environments.

## 3. Computational Method

Based on the Monte Carlo simulation of 10 parallel monoatomic carbon chains in the form of a hexagonal array in Figure 3, the distribution of the bond angles will be computed in a series of chain lengths in the presence of weak van der Waals’ force via the Hamiltonian *H* below.

$$H=e^{-T/T_{bj}}\sum_{m=1}^{M}\sum_{n=1}^{N-1}\left|E_{m,n,j}e^{-\frac{\left(r_{m,n}-r_{m,n,j}^{eq}\right)}{0.5r_{m,n,j}^{eq}}}-E_{2}\right|+e^{-T/T_{bj}}\sum_{m=1}^{M}\sum_{n=1}^{N-1}J_{A}{\left(\cos\theta +1\right)}^{2}-4\varphi \left[\sum_{n,m}^{}{\left(\frac{\sigma }{r}\right)}^{6}-{\left(\frac{\sigma }{r}\right)}^{12}\right]$$

where M, N, $E_{2}$, and T are the total number of chains, the total number of carbons in each chain, double bond energy, and temperature, respectively. The formation of single, double, and triple bond $E_{m,n,1}$, $E_{m,n,2}$, and $E_{m,n,3}$ corresponds to j=1,2, and 3, respectively [12]. The energies of single, double, and triple bonds at a temperature of 300 K are 348 kJ/mol, 614 kJ/mol, and 839 kJ/mol, respectively [12]. The r is computed in Cartesian coordinates and $r_{m,n,j}^{eq}$ is the equilibrium position. The C−C, C=C, and C≡C bond distances are $r_{m,n,1}^{eq}$ = 1.54 Å, $r_{m,n,2}^{eq}$ = 1.34 Å, and $r_{m,n,3}^{eq}$ = 1.20 Å, respectively. For example, $r_{4,20,3}^{eq}$ refers to the equilibrium position of the 20th atom along the 4th chain, which is connected by a triple bond. The temperature to break the covalent bond $T_{bj}$ is determined by $E_{j}=k_{B}T_{bj}$; the Boltzmann constant equals $k_{B}=1.38\times 10^{-23}JK^{-1}$. The van der Waals energy $E_{vdw}^{}$ is the only interaction between the adjacent carbon chains with sample length $\tau_{s}$, φ and σ are $8.1\times 10^{-23}\;J$ and $1.23\times 10^{-10}\;m$, respectively [11,12]. The van der Waals distance d is 0.3 nm unless otherwise specified. Three adjacent carbon atoms form one pivot angle with the appearance of kink structure, where the pivot angle in the linear carbon chain is defined as θ. The angular energy $J_{A}$ is set to 600 kJ/mol [12]; however, the effective angular energy in the system is weakened by the cosine term in the Hamiltonian. Carbon atoms interact with the nearest neighbors along the longitudinal direction. The carbon chain carrying N atoms is named N-CNA. For example, a carbon nanowire made up of 50 atoms is named 50-CNA.

In our model, all carbons are initially connected with double bonds and spaced 1.34 Å apart from each other. At each Monte Carlo step, the carbon atom is randomly selected and its coordinate can be changed, resulting in a variation of the van der Waals energy and a change in the types of covalent interactions during the iterations. At each temperature, the kinematics of the selected carbon, with atomic mass M, are governed by the formula $dz=\pm p\tau \sqrt{\frac{k_{B}T}{M}}$. The scattering time of a carbon atom is approximately ~2 × 10^−13^ sec, which is calculated by considering the root mean square velocity of the atom along the Z-axis within one period of motion in a 1D harmonic oscillator [12]. The frictional factor p, which ranges from 0.01 to 0.99, is used to represent the stochastic nature of collisions, where the rate of collision always varies from one place to another. If the random number R_z_ is greater than 0.5, the sign of dz is positive. Conversely, if R_z_ is less than or equal to 0.5, the sign of dz is negative. The sign of dx and dy depend on their respective random numbers R_x_ and R_y_; if R_x_ is greater than 0.5, dx is positive, and if R_y_ is greater than 0.5, dy is positive. Otherwise, R_x_ and R_y_ are negative. For the carbon chain, the amplitude of transverse vibration is smaller than that of longitudinal vibration and hence $dx=dy=\frac{k_{B}T}{(E_{m,n,1}+E_{m,n,2}+E_{m,n,3})/3}dz$.

We use another random number R_bond_ between 0 and 1 to control the trial type of covalent bonds at the trial stages:

If C−C bond is detected, it is switched to either C=C (R_bond_ > 0.5) or C≡C(R_bond_ ≤ 0.5).

If C=C bond is detected, it is amended to either C−C (R_bond_ > 0.5) or C≡C(R_bond_ ≤ 0.5).

If C≡C bond is detected, it is changed to either C=C (R_bond_ > 0.5) or C−C(R_bond_ ≤ 0.5).

When temperature increases, the type of covalent bond between atoms may also change depending on the energy difference (E_diff_) between the initial and trial Hamiltonian monitored by the Boltzmann factor $e^{\frac{-E_{diff}}{k_{B}T}}$ [12]. If the random number R_B_ between 0 and 1 is smaller than the Boltzmann factor, the selected carbon will accept the trial states. The Monte Carlo simulation is repeated 100,000 times at each temperature, unless otherwise specified.

## 4. Conclusions

In summary, kink structures in free-standing monoatomic carbon nanowire confer the potential material for chemisorption, which is an active area of research due to the issue of thermally impacted chemisorption at high temperatures. Through Monte Carlo simulations focusing on multi-monoatomic carbon chains laterally interacting with van der Waals forces, we studied the effects of the chain length and temperature on the kink structures. Our results suggest that at room temperatures, the nanowires maintain almost all their linearity regardless of chain length. Disordered kink structures are seen very obviously in shorter carbon chains above the Peierls transition temperature, which may enhance chemisorption at high temperatures. We postulate that any physical properties of the finite-length carbon chain predicted by ab initio calculations should take account of thermal instability at high temperatures. The development of such a system would create an opportunity to improve the prediction of the physical properties of carbon nanowires in high-temperature environments. Ultimately, it will be a valuable mission to investigate the thermally induced kink structures in the carbon nanowire that may be used for chemisorption in the future.

## Data Availability

The data can be shared upon reasonable request.
